# U-shaped association between plasma C-peptide and sarcopenia: A cross-sectional study of elderly Chinese patients with diabetes mellitus

**DOI:** 10.1371/journal.pone.0292654

**Published:** 2023-10-20

**Authors:** Ming-Jun Chen, Jing Leng, Jian-Ping Ni, Ai-Ling Xiong, Man-Yun Hu

**Affiliations:** Department of Endocrinology, Guiyang Fourth People’s Hospital, Guiyang, Gui Zhou Province, China; University of Campania Luigi Vanvitelli: Universita degli Studi della Campania Luigi Vanvitelli, ITALY

## Abstract

Limited research exists regarding the relationship between fasting plasma C-peptide levels and sarcopenia. As a result, our study aimed to examine this association in elderly Chinese diabetic patients. This cross-sectional study included 288 elderly patients with diabetes mellitus from the Fourth People’s Hospital in Guiyang who were enrolled prospectively between March 2020 and February 2023. The independent variable of interest was fasting plasma C-peptide, while the dependent variable was sarcopenia. Data on several covariates, including demographic factors, lifestyle habits, co-morbidities, anthropometric indicators, and laboratory indicators, were also collected. Of the 288 participants, 27.43% (79/288) had sarcopenia. After adjusting for potential confounding variables, we found a U-shaped association between fasting plasma C-peptide levels and sarcopenia, with inflection points identified at approximately 774 pmol/L and 939 mmol/L. Within the range of 50–744 pmol/L, each 100 pmol/L increase in CysC was associated with a 37% decrease in the odds of sarcopenia (odds ratio [OR], 0.63; 95% confidence interval [CI], 0.49 to 0.83; P < 0.001). Additionally, within the range of 939–1694 pmol/L, each 100 pmol/L increase in fasting plasma C-peptide was associated with a 76% increase in the odds of sarcopenia (odds ratio [OR], 1.76; 95% confidence interval [CI], 1.11 to 2.81; P = 0.017). Our study revealed a U-shaped association between fasting plasma C-peptide levels and the likelihood of sarcopenia, with lower risk in the range of 774–939 pmol/L. These findings may assist in the development of more effective prevention and treatment strategies for sarcopenia in elderly diabetic patients.

## Introduction

Sarcopenia is a condition where there is progressive loss of skeletal muscle mass and function with age, caused by a decrease in the number of muscle fibers and impaired muscle remodeling due to reduced satellite cell activation and proliferation capacity [1, 2]. The prevalence of sarcopenia is rising, affecting around 25% of people under 70 years old and 40% of those aged 80 years or older [3]. In China, the prevalence of sarcopenia among adults above 65 years old is 15% [4]. As the aging population continues to grow, this number is expected to increase in the future [5]. Sarcopenia poses significant health risks for older adults, including reduced physical activity and increased incidence of falls and fractures [6]. Effective pharmacological treatments for sarcopenia are currently lacking, making it a major public health concern [7].

Insulinogenic ligand peptide, also known as C-peptide, was initially considered a biologically insignificant byproduct but later found to affect various physiological complications associated with diabetes such as neuropathy, nephropathy, and encephalopathy [8, 9]. A recent animal study also showed that c-peptide administration modulates body composition and protects skeletal muscle mass from type 1 diabetes-induced atrophy [10].

However, there is limited evidence on the association between C-peptide and sarcopenia. Since C-peptide is associated with insulin, which plays an important role in maintaining metabolic homeostasis in the body, including metabolic regulation of muscle tissue [11], and the prevalence of skeletal sarcopenia was significantly higher in elderly diabetic patients than in non-elderly diabetic patients [12], we hypothesized that there might be an association between C-peptide and skeletal sarcopenia in elderly diabetic patients. Thus, we propose a single-center cross-sectional study to observe the association between fasting C-peptide and skeletal sarcopenia in elderly diabetic patients above 65 years old.

## Participants and methods

### Study design

This is a cross-sectional study conducted by the Endocrine Care Center of the Fourth People’s Hospital in Guiyang, China. In 2021, a health management program was launched for elderly patients with diabetes. The aim of this program is to evaluate the health condition of elderly diabetic patients through a series of physical examinations and health monitoring, thereby preventing the development of chronic complications such as diabetic nephropathy, diabetic foot, and diabetic retinopathy. Additionally, the program includes monitoring for sarcopenia.

### Study population

**Fig 1:**
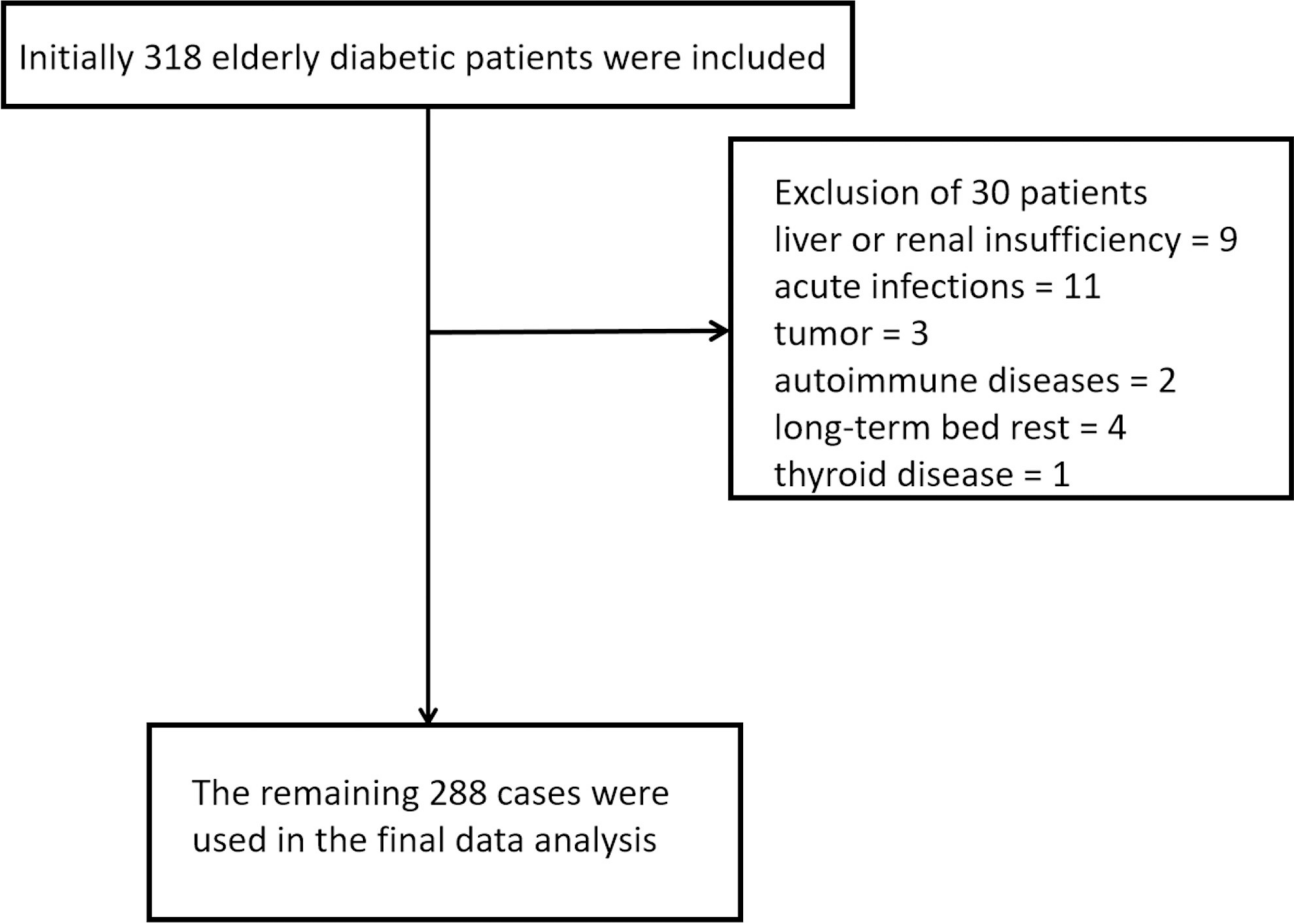
The process of enrolling patients involved in this study.

This study prospectively enrolled patients who participated in a health management program for older patients with diabetes at our endocrine care center from May 2021 to February 2023. The patient inclusion process was non-selective and consecutive. The diagnosis of type-2 diabetes mellitus was based mainly on the Chinese Diabetes Control Guidelines 2020 Edition published by the Chinese Diabetes Association. The inclusion criteria were: (1) age greater than or equal to 65 years, (2) clear history or diagnosis of type 2 diabetes. Exclusion criteria were: (1) age younger than 65 years, (2) liver or renal insufficiency, (3) various types of acute infections in combination, (4) tumors, (5) thyroid disease, (6) autoimmune diseases, (7) combined connective tissue diseases, (8) acute myocardial infarction, (9) acute cerebral infarction and cerebral hemorrhage, (10) type 1 diabetes, diabetic nephropathy or hyperosmolar coma, (11) long-term bed rest, local braking or hemiplegia. This study was approved by the Ethics Committee of the Fourth People’s Hospital (approval number: [2021]No 005). All patients and their blood relatives were informed in detail about the health management program for elderly diabetic patients, signed informed consent forms, and agreed to use their clinical information and measurement data for data analysis under the premise of anonymisation of personal privacy. Initially, 318 elderly diabetic patients were enrolled, and based on a series of nadir criteria, 288 remained for final data analysis. The specific screening process is detailed in Fig 1.

### Variable

#### Outcome variable

The outcome variable in this study was the presence or absence of sarcopenia, which was recorded as a dichotomous variable (yes/no) in the database. The diagnosis of sarcopenia was based on the criteria defined by the Asian Working Group on Sarcopenia Consensus 2019 (AWGS2019) [13], which describes sarcopenia as a condition characterized by low skeletal muscle mass accompanied by low hand grip strength, decreased gait speed, or both. We mainly referred to the cut-off values provided by AWGS2019 for the diagnosis of sarcopenia. Specifically, we used the diagnostic cut-off values of <7.0 kg/m2 for males and <5.4 kg/m2 for females for low skeletal muscle mass, as defined by skeletal muscle index (SMI). For low muscle strength, we used handgrip strength values of <28.0 kg for males and <18.0 kg for females. Additionally, a decrease in gait speed to <1.0 m/s was defined as decreased gait speed. Skeletal muscle mass, muscle strength, and somatic function were measured according to the details provided in S1 Table in S1 File.

#### Independent variable of interest

The independent variable of interest in this study was fasting C-peptide, which was recorded as a continuous variable in the database. All patients fasted for 8 hours, and venous blood was drawn from the elbow at 8:00am and sent to the central laboratory of the Fourth People’s Hospital in Guiyang for testing (Roche Automated Chemistry Analyzer (machine model: E601, Manufacturer: Roche). The testers were blinded to the subjects’ sarcopenia status at the time of the test.

#### Covariates

The selection of covariates was mainly based on previous literature that studied the risk factors for sarcopenia and the clinical experience of the investigators. The covariates used in this study included: (1) socio-demographic variables such as gender (male/female), age (continuous variable in years), literacy (illiterate + primary/junior high/high school and above), and marital status (married/other (divorced, widowed)); (2) anthropometric indicators such as body mass index (BMI) [14] and fat mass [15]; (3) Barthel Index score [16, 17]; (4) lifestyle habits such as smoking (current smoker/non-smoker/ex-smoker) and drinking status (current drinker/non-drinker/ex-drinker); (5) co-morbid history including hypertension, hyperlipidemia, cardiovascular disease, osteoporosis, and chronic obstructive pulmonary disease (COPD); and (6) other indicators such as hemoglobin [18], glycated hemoglobin, and globulin [19].

### Missing data addressing

Because the percentage of missing data in this study was less than 5%, no multiple imputation was employed (S2 Table in S1 File).

### Statistical analysis

We summarized continuous variables using means and standard deviations, and categorical variables using numbers and proportions. Additionally, we categorized participants into three groups based on their fasting c-peptide tertiles and used chi-square tests for categorical variables and one-way ANOVA for continuous variables to analyze differences between the three groups. Multivariable logistic regression was used to investigate the relationship between fasting c-peptide and sarcopenia. We displayed model 1 with no adjusted covariates, model 2 which was only adjusted for age, sex, education level and marital status, and model 3 which was adjusted for all covariates presented in Table 1 simultaneously. We divided the fasting C-peptide by 100 to facilitate observation of changes in OR. Therefore, the results should be interpreted per 100 unit change.

**Table 1:** Baseline characteristics of elder patients with diabetes categorized by fasting C-peptide (tertile).

	Non-myasthenia gravis	Sarcopenia	P-value
**N**	209	78	
**BMI, mean±sd, (kg/m2)**	24.30 ± 3.06	23.10 ± 2.72	0.003
**Fat mass, mean±sd**	18.36 ± 7.22	20.46 ± 6.63	0.026
**Diabetes duration time, mean±sd, year**	9.77 ± 7.44	11.64 ± 5.64	0.045
**Glycated hemoglobin, mean±sd, %**	8.82 ± 2.62	9.14 ± 2.54	0.354
**Hemoglobin, mean±sd, g/L**	141.44 ± 17.72	136.06 ± 16.60	0.021
**Globulin, mean±sd, g/L**	30.07 ± 4.92	30.86 ± 5.77	0.250
**Fasting C-peptide, mean±sd, pmol/L**	766.09 ± 316.90	714.18 ± 435.50	0.269
**Age, mean±sd, year**	69.25 ± 6.06	71.12 ± 6.75	0.025
**Bartel Index, mean±sd**	96.62 ± 6.70	90.75 ± 10.91	<0.001
**Sex, No (%)**			0.233
**Male**	83 (39.71%)	25 (32.05%)	
**Fmale**	126 (60.29%)	53 (67.95%)	
**Education level, No (%)**			0.710
**Primary + illiterate**	34 (17.62%)	13 (18.31%)	
**junior high school**	84 (43.52%)	27 (38.03%)	
**High school and above**	75 (38.86%)	31 (43.66%)	
**Marital status, No (%)**			0.843
**Married**	152 (74.51%)	56 (75.68%)	
**Living alone (divorced, widowed)**	52 (25.49%)	18 (24.32%)	
**Hypertension history, No (%)**			0.028
**No**	144 (70.24%)	44 (56.41%)	
**Yes**	61 (29.76%)	34 (43.59%)	
**Hyperlipidemia, No (%)**			0.937
**No**	53 (25.85%)	20 (26.32%)	
**Yes**	152 (74.15%)	56 (73.68%)	
**History of osteoporosis, No (%)**			0.035
**No**	148 (70.81%)	45 (57.69%)	
**Yes**	61 (29.19%)	33 (42.31%)	
**COPD history, No (%)**			0.247
**No**	92 (44.88%)	41 (52.56%)	
**Yes**	113 (55.12%)	37 (47.44%)	
**Smoking status, No (%)**			0.019
**Non-smoker**	128 (65.31%)	36 (46.75%)	
**Current smoker**	54 (27.55%)	32 (41.56%)	
**Smoking cessation**	14 (7.14%)	9 (11.69%)	
**Alcohol consumption status, No (%)**			<0.001
**Never drank alcohol**	160 (80.81%)	43 (55.13%)	
**Drinking alcohol**	13 (6.57%)	19 (24.36%)	
**Quit drinking**	25 (12.63%)	16 (20.51%)	
**Cardiovascular disease, No (%)**			0.098
**No**	106 (50.72%)	31 (39.74%)	
**Yes**	103 (49.28%)	47 (60.26%)	

Note: The difference between the sample sum of each column and the total number shown in Table 1 is due to missing data

Abbreviation:

BMI: body mass index

SD: Standard deviation

COPD: Chronic Obstructive Pulmonary Disease

As logistic regression is not well-suited for analyzing non-linear associations, we utilized a generalized additive model (GAM) to investigate the relationship between fasting C-peptide and sarcopenia. Initially, we smoothed the curve to observe the true association between fasting C-peptide and sarcopenia, and then determined the inflection point values using a recursive algorithm. Finally, effect values (Odds ratio) and 95% confidence intervals for fasting C-peptide and sarcopenia were calculated using a two-piecewise linear model on both sides of the inflection point. To ensure the robustness of our findings, we conducted a series of sensitivity analyses: (1) Fasting C-peptide was divided into three categories and P for trend was calculated to observe the robustness of the association between fasting C-peptide as a continuous variable and as a categorical variable with sarcopenia. (2) We used a log-likelihood ratio test to determine whether a two-piecewise linear model or a logistic regression model more accurately fit the association between fasting C-peptide and sarcopenia. All analyses were performed using the statistical packages R Foundation (http://www.R-project.org) and EmpowerStats (http://www.empowerstats.com,X&Y Solutions, Inc., Boston, MA). Statistical significance was defined as a two-sided p-value less than 0.05.

## Results and discussion

### The baseline characteristics of Chinese HF patients

We analyzed and compared the distribution of patient characteristics between the sarcopenic and non-sarcopenic groups. The results are presented in Table 1. Our study included elderly diabetic patients, with a mean age of 69.74 ± 6.28 years, and a male-to-female ratio of 1:1.6, with males accounting for 37.5% (108/288) of the total patients. Of the patients, 27.43% (79/288) were found to have sarcopenia. Compared to non-sarcopenic patients, those in the sarcopenic group had a lower body mass index, fasting plasma C-peptide levels, hemoglobin levels, and Bartel Index (indicating poorer physical function). In contrast, elderly diabetic patients in the sarcopenic group had higher fat mass, duration of diabetic load, combined cardiovascular disease, hypertension, diabetes mellitus, osteoporosis, smoking and alcohol consumption compared to the non-sarcopenic group. higher. There were no statistically significant differences in HbA1C levels, globulin, education, gender, marital status, hyperlipidemia, and COPD between the two groups.

### The association between fasting C-peptide and sarcopenia obtained from logistic regression model, both univariate and multivariable

We investigated the association between fasting C-peptide and sarcopenia using different covariate adjustment strategies (Table 2). In model 1 (the unadjusted model), the results indicated that there was no significant correlation between fasting C-peptide and the prevalence of sarcopenia (odds ratio [OR], 0.92; 95% confidence interval [CI], 0.85 to 1.00; P = 0.065). The same results were obtained from model 2. Even after adjusting for sex, age, education level, and marital status, we still did not observe a substantial association between fasting C-peptide (per 100 pmol/L) and sarcopenia (odds ratio [OR], 0.92; 95% confidence interval [CI], 0.84 to 1.01; P = 0.085). When we adjusted for all the covariates listed in Table 1 (model 3, fully-adjusted model), we found a 4% decrease in the probability of sarcopenia for each 100 pmol/L increase in fasting C-peptide. Nevertheless, due to the wider distribution of confidence intervals, we could not reject the null hypothesis (odds ratio [OR], 0.96; 95% confidence interval [CI], 0.85 to 1.07; P = 0.442).

**Table 2:** Associations between fasting C-peptide and sarcopenia using logistic regression models.

	model 1 OR, 95%CI, P value	model 2 OR, 95%CI, P value	model 3 OR, 95%CI, P value
**Sarcopenia (Yes/No)**			
**Fasting C-peptide (continuous variable)**	0.92 (0.85, 1.00) 0.065	0.92 (0.84, 1.01) 0.085	0.96 (0.85, 1.07) 0.442
**Fasting C-peptide (grouped by tertile)**			
**Low**	Reference	Reference	Reference
**Middle**	0.44 (0.23, 0.84) 0.012	0.37 (0.18, 0.75) 0.006	0.38 (0.16, 0.93) 0.035
**High**	0.54 (0.29, 1.02) 0.057	0.58 (0.29, 1.13) 0.107	0.71 (0.29, 1.72) 0.443
**P for trend**	0.056	0.093	0.418

Abbreviation:

OR: odds ratio

CI: Confidence interval

model 1: No covariates were adjusted for

model 2: Adjusted for age, sex, education level and marital status

model 3: Adjusted for all covariates presented in Table 1.

To conduct a sensitivity analysis, we transformed fasting C-peptide into a categorical variable based on tertile and assessed whether the outcomes remained consistent when fasting C-peptide was used as either a categorical or continuous variable (Table 2). The p-values of the trend test for the three models were 0.056 (model 1), 0.093 (model 2), and 0.418 (model 3), respectively. These outcomes were consistent with the findings when fasting C-peptide was applied as a continuous variable.

### The nonlinearity addressing

**Fig 2:**
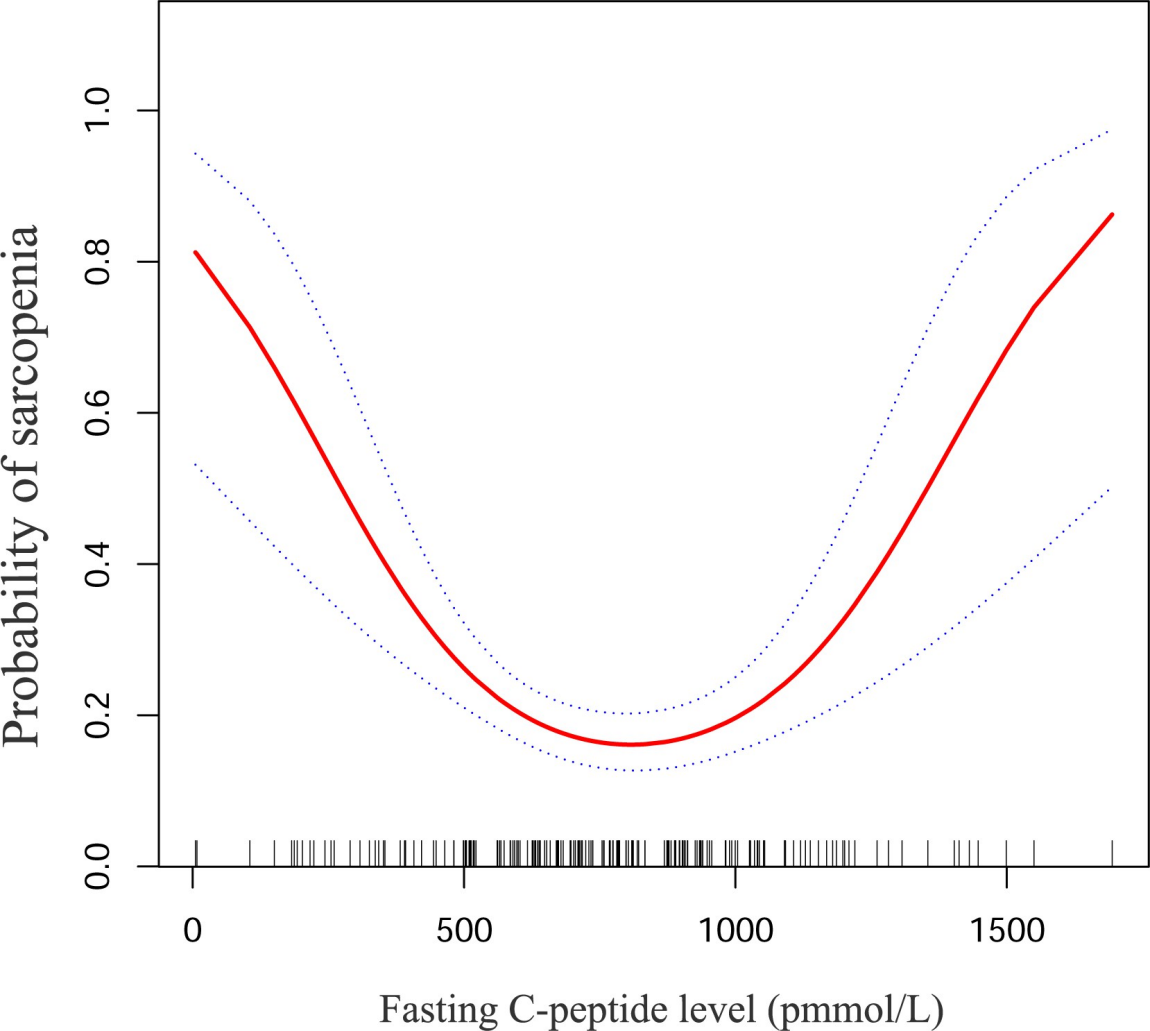
A non-linear relationship between fasting C-peptide and sarcopenia. The level of fasting C-peptide is plotted on the horizontal axis, while the probability of sarcopenia is plotted on the vertical axis. The middle line reflects the trend in the association between C-peptide and sarcopenia, while the upper and lower lines indicate the corresponding 95% confidence intervals.

To investigate the potential nonlinear relationship between fasting C-peptide and sarcopenia, we utilized a GAM model and smoothed curve fitting to reveal a U-shaped correlation between these two variables (Fig 2). To better explicate this U-shaped curve, we applied a recursive algorithm to calculate the inflection points of fasting C-peptide, which were determined to be 744 and 939 pmol/L. Thereafter, we deployed a two-piece linear model to estimate the effect values and confidence intervals on both sides of the inflection points (Table 3). Our results showed that for every 100 pmol/L increase in fasting C-peptide within the range of 50–744 pmol/L, there was a tendency for the probability of sarcopenia to decrease by 37% (odds ratio [OR] 0.63; 95% confidence interval [CI] 0.49 to 0.83; P < 0.001). On the contrary, we did not observe a substantial association between fasting c-peptide and the probability of sarcopenia when fasting c-peptide was within the 744–939 pmol/L interval (odds ratio [OR] 1.27; 95% confidence interval [CI] 0.34 to 4.59; P = 0.720). However, when fasting C-peptide was in the 939–1694 range, each 100 pmol/L increase in fasting C-peptide was associated with a 76% increase in the probability of sarcopenia (odds ratio [OR] 1.76; 95% confidence interval [CI] 1.11 to 2.81; P = 0.017)). Additionally, upon comparing the logistic regression model fit with the two-piece linear model fit, the log likelihood ratio yielded a P value of less than 0.001, indicating that the two-piece linear model provided a better fit for the association between fasting C-peptide and sarcopenia.

**Table 3:** Exploration of nonlinear association between fasting C-peptide and sarcopenia using two-piecewise linear model.

	Probability of sarcopenia OR, 95%CI, P value
**Fitting model by standard binary logistic regression model (per 100 change of fasting C-peptide)**	0.96 (0.85, 1.07) 0.442
**Fitting model by two-piecewise linear model (per 100 change of fasting C-peptide)**	
**Inflection point (Actual value of fasting C-peptide / 100)**	7.74 and 9.39
**≤ 7.74**	0.63 (0.49, 0.83) 0.0007
**>7.74 and ≤ 9.39**	1.27 (0.35, 4.59) 0.7203
**> 9.39**	1.76 (1.11, 2.81) 0.0173
**P for log likely ratio test**	<0.001

We conducted a single-centre cross-sectional study in an elderly Chinese diabetic population to investigate the association between fasting plasma C-peptide and sarcopenia. The results showed that both low and high fasting C-peptide levels were associated with a higher probability of sarcopenia, indicating a U-shaped association between C-peptide and sarcopenia. We observed that fasting C-peptide was associated with a lower probability of sarcopenia within the range of 774 to 939 pmol/L.

Previous evidence on the association of fasting C-peptide with sarcopenia is insufficient. Lu YX et al [20]. found a negative association between fasting C-peptide and the risk of sarcopenia in 41 diabetic patients and 205 non-diabetic patients, but their study only included 41 diabetic patients and diabetes status was only adjusted as a covariate. In addition, they only adjusted for sex, age, diabetes status, and total body fat mass. Our study explored possible non-linear associations and found negative associations between fasting C-peptide and sarcopenia in the 50–774 pmol/L range, which is partially consistent with the results of Hitomi Miyake et al [21]. and Tanaka K et al [22]. Hitomi Miyake et al. found that fasting C-peptide values were significantly higher in patients in the high skeletal muscle content group than in the low skeletal muscle content group in a study on the association between low skeletal muscle content and all-cause mortality in a diabetic population. Tanaka K et al. similarly found a negative association between C-peptide and sarcopenia in 191 men with type 2 diabetes but did not explore non-linear associations.

In contrast to earlier findings, our study discovered a link between high fasting C-peptide levels (939 pmol/L–1700 pmol/L) and sarcopenia in elderly diabetes patients, which we believe is the first of its kind. We propose many explanations for this unusual U-shaped association trend: First, C-peptide is an insulin precursor that, unlike insulin, is filtered by the kidneys and eliminated in the urine rather than being metabolized by the liver. As a result, monitoring C-peptide levels can reflect islet cell function without being influenced by external influences such as food [23]. Low fasting C-peptide levels may indicate insulin production and release inhibition, resulting in metabolic inefficiency, decreased muscle mass, and an increased risk of sarcopenia [24]. Previous research has shown that insulin is a key regulator of glucose absorption and use in peripheral tissues, including muscle [25]. Inadequate insulin secretion and release caused by low C-peptide levels can result in diminished insulin signaling and impaired glucose metabolism, resulting in decreased amino acid absorption and protein synthesis in muscle, resulting in decreased muscle mass and an increased risk of sarcopenia [25, 26]. Furthermore, low insulin levels stimulate glucagon secretion, which causes muscle proteolysis and amino acid release into the circulation, increasing the risk of sarcopenia [27]. High fasting C-peptide levels, on the other hand, may indicate insulin resistance [28, 29]. As a result, increased insulin signaling, particularly in peripheral tissues, might result in decreased muscle mass. Sarcopenia is caused by three mechanisms: (1) increased proteolytic metabolism and decreased protein synthesis in skeletal muscle; (2) increased expression of the FoxO family, which either directly or indirectly attenuates skeletal muscle; and (3) autophagy in skeletal muscle cells [29]. Furthermore, Sara Manrique-Arija et al [30]. discovered that insulin resistance was associated with higher levels of CRP and interleukin-1 in a cross-sectional study of arthritis patients, whereas previous literature confirmed that muscle atrophy is a major determinant of vulnerability in old age and is associated with high levels of inflammation [31]. Thus, despite the fact that this study was merely cross-sectional in nature, the association between high C-peptide, insulin resistance, inflammation, and muscle atrophy may be the cause of the positive correlation between these variables. To establish definitive evidence, additional high-level investigations, including cohort studies and randomized controlled clinical trials, are required. Second, high levels of C-peptide point to poorly controlled diabetes. On the other hand, prior clinical research indicates that vulnerability in older people is frequently linked to inadequate chronic disease management [32]. As a result, this may also be one of the possible causes of the positive association between high C-peptide levels and muscle atrophy. Lastly, 25-hydroxyvitamin D3 in elderly people is frequently linked to muscular atrophy [33]. We used the cut points of 7.74 and 9.39 to divide the population into three categories. The levels of 25-hydroxyvitamin D3 were substantially greater in the two low C-peptide groups (7.74 and 7.74–9.39) than in the high C-peptide group (>9.39), according to our research (S1 Fig in S1 File). Therefore, the considerable drop in 25-hydroxyvitamin D3 levels may potentially be linked to the positive connection between a high C-peptide group and muscle atrophy.

Our study reports a U-shaped association between fasting C-peptide levels and the risk of sarcopenia, which, to our knowledge, has not been previously reported in the literature. Our findings suggest that in elderly diabetic patients, maintaining appropriate C-peptide levels is crucial for normal metabolism and muscle mass and may prevent the development of sarcopenia. While our study provides relevant clinical data for future research on this topic, we acknowledge that cross-sectional evidence is relatively weak and further studies, such as prospective cohort studies, are required for validation. We continue to follow up with elderly people with diabetes mellitus but without concomitant sarcopenia in our center, and as such, our findings have important implications for future research on this topic. By utilizing easily accessible, simple, and inexpensive assessments such as fasting C-peptide levels, healthcare professionals can identify patients at risk of sarcopenia early on and take preventive measures accordingly. In summary, our study supports the notion that monitoring C-peptide levels in elderly diabetic patients is important for maintaining normal metabolism and muscle mass, thus avoiding the development of sarcopenia. We hope that our findings will promote further research in this area and eventually lead to the adoption of more effective prevention and treatment strategies for sarcopenia.

Our study has several strengths that contribute to its validity. Firstly, we utilized advanced algorithms (GAM model and two-piece linear model) which allowed us to effectively detect a true association between fasting C-peptide levels and the risk of sarcopenia in elderly Chinese diabetics. Secondly, we conducted a series of sensitivity analyses and utilized different variable tuning strategies, reducing the possibility that our findings were merely incidental and increasing the robustness of our results. Thirdly, our study’s prospective, continuous, and non-selective inclusion of patients, combined with the application of blinding to reduce possible distortion of results due to selection bias and observation bias.

However, our study does have some limitations that must be taken into account when interpreting our findings. Firstly, the study population was limited to Chinese diabetics and caution should be taken when extrapolating our findings to other races. Secondly, our results are only applicable to diabetic patients over 65 years of age and cannot be generalized to other populations (e.g., type 1 diabetes, hepatic and renal insufficiency, tumor patients, autoimmune system diseases, etc.). Thirdly, as an observational cross-sectional study, our findings are subject to confounding, although we have rigorously adjusted for confounders and assessed the robustness of our results through sensitivity analysis. Fourthly, the nature of the cross-sectional study design limits our ability to assess causality; we can only observe associations. Finally, we can only adjust for measurable confounders, making it impossible to adjust for unmeasurable confounding factors. Therefore, future clinical studies with higher levels of evidence in larger populations are warranted to validate our findings.

## Conclusion

In conclusion, our study found that the association between fasting plasma C-peptide levels and the risk of sarcopenia is U-shaped. Specifically, fasting plasma C-peptide was associated with a lower risk of sarcopenia when in the range of 774–939 pmol/L. We hope that our findings will contribute to the development of more effective prevention and treatment strategies for sarcopenia in elderly diabetic patients.

## Supporting information

S1 Checklist STROBE statement—checklist of items that should be included in reports of observational studies. (DOCX)

S1 File The "Supporting information" as a whole file contains two tables and one figure. They are listed separately as follows: S1 Fig. The distribution of 25-hydroxyvitamin D3 in different Group of C-peptide. The y-axis represents the levels of 25-hydroxyvitamin D3. The different modes of bars represent different C-peptide groups. The error bars represent the standard deviation. S1 Table: The measurement of Skeletal muscle mass, muscle strength, and somatic function. The first column of the table lists the names of the test indicators, and the second column lists the corresponding test methods and procedures. S2 Table: The description of missing data. The first column represents variable names, the second and third columns represent the number of missing and non-missing samples, respectively, and the fourth column represents the proportion of missing values.
(DOCX)

S2 File (PDF)

S3 File (PDF)

S1 Data (XLS)
