# Supplementary material for: U-shaped association between plasma C-peptide and sarcopenia: A cross-sectional study of elderly Chinese patients with diabetes mellitus
Source: PLoS One. 2023 Oct 20;18(10):e0292654. doi: 10.1371/journal.pone.0292654 (PMC10588858; doi:10.1371/journal.pone.0292654)

Supplemental table 1：The measurement of Skeletal muscle mass, muscle strength, and somatic function

| Characteristics |  |
| --- | --- |
| Skeletal muscle mass | body composition assessment using CT and MR scans is considered the gold standard. These scans provide precise anatomical detail, especially when assessing skeletal muscle volume. Additionally, they are the only examinations capable of direct assessment of visceral and subcutaneous fat content. In this study, CT scans were used to measure skeletal muscle mass. CT measurements were taken on the abdomen, thighs, and upper arms, most commonly at the level of the third lumbar vertebra. Cross-sectional area (CSA) and skeletal muscle index (SMI), which is the ratio of CSA to height squared, were measured. Semi-automatic contouring was performed using third-party software based on CT values, and skeletal muscle edges were manually trimmed by the investigator before the software calculated the intra-contour area. |
| muscle strength | grip strength is used as an indicator of muscle strength according to the AWGS2019 standard. In Asia, the most commonly used device for measuring grip strength is the spring-loaded grip, followed by the hydraulic grip. The AWGS2019 recommends using both devices for the diagnosis of sarcopenia; however, direct comparison of results measured with different devices is not recommended, as older people may have higher results with the hydraulic grip than with the spring-loaded grip. In this study, grip strength was measured using a spring-loaded grip strength device in the standing position with the elbow extended. If the older person was unable to stand independently, the sitting position was chosen. For the diagnosis of sarcopenia, the AWGS2019 recommends a grip strength diagnostic threshold of <28.0kg for men and <18.0kg for women. |
| somatic function | the AWGS2019 recommends using the SPPB, 6m gait speed, and 5 sit-up tests for the assessment of somatic function. The AWGS2019 standardizes the measurement of gait speed as the time taken to walk 6m at a normal gait speed from the start of movement, without acceleration or deceleration, taking at least two measurements and recording the average speed. The AWGS2014 recommends a diagnostic threshold of 0.8 m/s for sarcopenia gait speed. In practice, many studies of frailty and disability have used a 6m gait speed <1.0m/s to reflect slow movement. The EWGSOP2018 recommends an SPPB score of ≤8 for decreased somatic function, but a systematic review including 17 studies (n=16534) found that an SPPB score of ≤9 was a better predictor of all-cause mortality. Therefore, the AWGS2019 recommends using an SPPB score of ≤9 as an indicator of decreased somatic function. In this study, a 6m gait speed was used as a measure of somatic function. |

Supplemental table 2: The description of missing data

| Variables | Without missing | Missing | Missing rate (%) |
| --- | --- | --- | --- |
| Sex | 288 | 0 | 0 |
| age | 288 | 0 | 0 |
| BMI | 288 | 0 | 0 |
| Education level | 275 | 13 | 4.51 |
| Bartel Index | 278 | 10 | 3.47 |
| Smoking status | 274 | 14 | 4.86 |
| Fat mass | 288 | 0 | 0 |
| Drinking status | 277 | 11 | 3.82 |
| Marital status | 279 | 9 | 3.125 |
| Hypertension history | 284 | 4 | 1.39 |
| Hyperlipidemia | 282 | 6 | 2.08 |
| Diabetes duration time | 288 | 0 | 0 |
| History of osteoporosis | 288 | 0 | 0 |
| Glycated hemoglobin | 288 | 0 | 0 |
| Hemoglobin | 288 | 0 | 0 |
| Cardiovascular disease | 288 | 0 | 0 |
| Globulin | 288 | 0 | 0 |
| COPD history | 288 | 0 | 0 |

Supplemental Figure 1: The distribution of 25-hydroxyvitamin D3 in different

Group of C-peptide


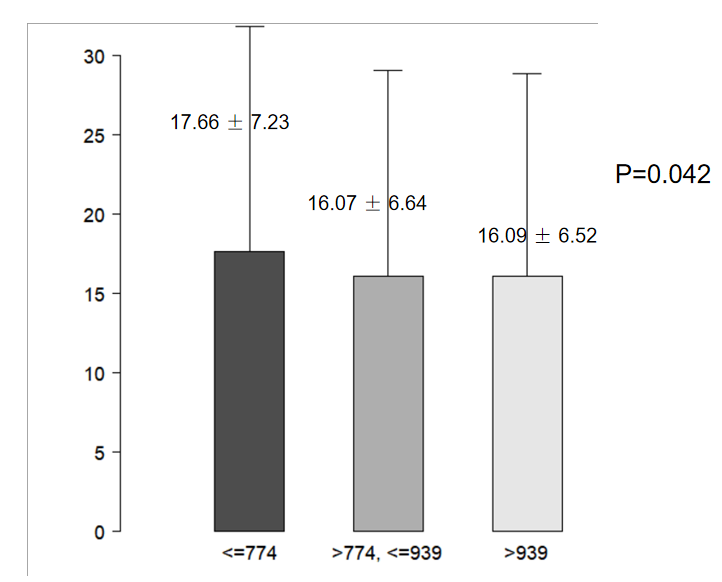

Supplement: S1 File — They are listed separately as follows: S1 Fig. The distribution of 25-hydroxyvitamin D3 in different Group of C-peptide. The y-axis represents the levels of 25-hydroxyvitamin D3. The different modes of bars represent different C-peptide groups. The error bars represent the standard deviation. S1 Table: The measurement of Skeletal muscle mass, muscle strength, and somatic function. The first column of the table lists the names of the test indicators, and the second column lists the corresponding test methods and procedures. S2 Table: The description of missing data. The first column represents variable names, the second and third columns represent the number of missing and non-missing samples, respectively, and the fourth column represents the proportion of missing values. (DOCX) [file pone.0292654.s002.docx]
