# Supplementary material for: U-shaped association between plasma C-peptide and sarcopenia: A cross-sectional study of elderly Chinese patients with diabetes mellitus
Source: PLoS One. 2023 Oct 20;18(10):e0292654. doi: 10.1371/journal.pone.0292654 (PMC10588858; doi:10.1371/journal.pone.0292654)
Supplement: S3 File — (PDF) [file pone.0292654.s004.pdf]

贵阳市第四人民医院医学伦理委员会
项目审查申请表

[2021]伦审第[005]号

项目名称	基于CT诊断的肌少症与老年2型糖尿病伴骨质疏松的相关性研究				
项目起止时间	2021年5月—2023年12月		申请科室	内分泌科、老干科	
项目类别	1.药物临床试验/医疗器械临床试验 2.医疗新技术/医学诊断试剂临床试验 3.涉及人的生物医学研究 4.动物实验				
申请类别	1.初始申请 2.复审申请 3.变更申请 4.暂停或终止申请 5.其他				
项目基本信息					
负责人姓名	胡曼云	学历/学位	大学本科	职称	主任医师
联系电话	13984375727				
主要研究方向	内分泌糖尿病、骨代谢				
主要研究人员	赵予、申红、何军、陈铭俊、李霞、熊爱玲、钟高晖				
合作研究单位	无				

项目摘要（初始、复审、变更、暂停或终止、其他情况说明）：

骨骼肌是人体运动最重要器官□随年龄增长，骨骼肌逐年减少□肌肉力量和肌肉质量中年后可随年龄增加而减少□研究显示，50岁以上人群的腿部肌肉力量每年减少1.5%-5.0%，肌肉质量每年减少1%-2%，且于70岁以后肌肉质量每十年可减少15%，最终可发展为肌少症□老年人身患肌少症后，行走、坐起、举物等日常活动受限，易造成无力、跌倒、骨折，进而引发心脑血管和呼吸等系统疾病，严重者导致失能，甚至死亡□因此，肌少症给家庭和社会带来严重问题。

糖尿病是全球患病率最高的慢性非传染性疾病之一，《中国糖尿病诊疗指南（2021版）》报道，60~69岁糖尿病患病率为28.8%，在≥70岁的人群中患病率为31.8%。社会老龄化与不断增长的老年糖尿病人群是我国社会面临的严峻考验，老年糖尿病患者合并的种慢性并发症，如骨质疏松症，更易跌倒、骨折等不良事件，增加家庭和社会的医疗负担。

糖尿病、骨质疏松与肌少症的发生发展有一定关系，糖尿病病理生理状态及并发症，肌肉收缩力学负荷对骨骼机械力的影响，骨骼与肌肉间内分泌调控的生物学机制都与肌肉功能

存在关联性。老年糖尿病伴骨质疏松患者合并肌少症，不仅受糖尿病、骨质疏松自身病理危害，而且受到肌少症不良影响，使其生活质量下降，导致多种不良后果□因此，研究老年糖尿病伴骨质疏松患者合并肌少症及肌功能减退的相关因素及不良影响显得尤为重要。			
审查方式	✓1.会议审查　2.简易程序审查　3.紧急情况受试者研究的审查　4.应急审查		
审查意见	✓1.同意开展　2.作必要的修正后同意开展　3.作必要的修正后重审<br>4.不同意开展　5.暂停或终止已批准的项目（试验）		
伦理委员会委员签名	[illegible] [illegible] [illegible] [illegible] 陈洪 [illegible] [illegible] [illegible] [illegible] [illegible]<br>贵阳市第四人民医院 医学伦理委员会		
主任委员签名	[illegible]	会议记录者签名	[illegible]
会议地点	行政楼三楼会议室	会议时间	2021. 4.2
保密声明：有关受试者的医学记录和研究资料保密。研究结果发表时，与受试者有关的信息资料不会被公开。			
